# High-throughput image-based plant stand count estimation using convolutional neural networks

**DOI:** 10.1371/journal.pone.0268762

**Published:** 2022-07-28

**Authors:** Saeed Khaki, Hieu Pham, Zahra Khalilzadeh, Arezoo Masoud, Nima Safaei, Ye Han, Wade Kent, Lizhi Wang

**Affiliations:** 1 Department of Industrial and Manufacturing Systems Engineering, Iowa State University, Ames, Iowa, United States of America; 2 College of Business, The University of Alabama in Huntsville, Huntsville, Alabama, United States of America; 3 Tippie College of Business, The University of Iowa, Iowa City, Iowa, United States of America; 4 Gies College of Business, The University of Illinois Urbana-Champaign, Champaign, Illinois, United States of America; 5 Syngenta Company, Slater, Iowa, United States of America; Fuzhou University, CHINA

## Abstract

The landscape of farming and plant breeding is rapidly transforming due to the complex requirements of our world. The explosion of collectible data has started a revolution in agriculture to the point where innovation must occur. To a commercial organization, the accurate and efficient collection of information is necessary to ensure that optimal decisions are made at key points of the breeding cycle. In particular, recent technology has enabled organizations to capture in-field images of crops to record color, shape, chemical properties, and disease susceptibility. However, this new challenge necessitates the need for advanced algorithms to accurately identify phenotypic traits. This work, advanced the current literature by developing an innovative deep learning algorithm, named DeepStand, for image-based counting of corn stands at early phenological stages. The proposed method adopts a truncated VGG-16 network to act as a feature extractor backbone. We then combine multiple feature maps with different dimensions to ensure the network is robust against size variation. Our extensive computational experiments demonstrate that our DeepStand framework accurately identifies corn stands and out-performs other cutting-edge methods.

## Introduction

The “phenotyping bottleneck” refers to the phenomenon that the agricultural community is not able to accurately and efficiently collect data on physical properties of crops in field [1]. This bottleneck is oftentimes due to resource limitations such as labor, time, and domain expertise in pathology and genetics, even for large scale farming operations and commercial breeding programs. A combination of systematic scalable technology and phenotyping is often referred to as high-throughput phenotyping (HTP). HTP is common in the commercial sector where resources are abundant due to the capabilities of large organizations to scale and purchase new technologies, but open-source use of these services is limited. These technologies accelerate efforts for screening numerous traits largely thanks to novel sensor technologies and computational improvements towards image-based phenotyping techniques [2]. Certain phenotypic traits such as early stage stand counting for corn (Zea *mays* L.) can only be accurately performed during its early growth stage. Applying an engineering system for early stage stand counts can assist in the collection of phenotypic data, ultimately, mitigating the phenotyping bottleneck. If this data collection time-window is missed, then the task is near impossible to complete [3].

Recognizing the data collection challenges facing modern agriculture, agronomists and researchers have turned to invest their resources into capturing images to be analyzed at a later date. Image-capturing devices like unmanned aerial vehicles, high definition cameras, and, even, cell phone cameras are being used as devices to collect information to be analyzed either live or at a later time [4, 5]. With these new technologies organizations and farmers are no longer bounded by the data collection time-window and now have the ability to manually analyze images at a later time. However, with this new approach comes additional problems such as storing massive amounts of image-based data and a familiar but new challenge—analyzing large scale image databases accurately and efficiently. To advance modern agriculture, new mechanisms should be made publicly available to agronomists that enable real-time decision making. The information contained within these images allows for timely management decision to optimize yield against harmful attack vectors (pests, diseases, drought, etc.).

To analyze large reserves of images quickly modern analytical tools have been invoked by various crops. Recent literature has seen the combination of planting phenotyping and traditional machine learning techniques (neural networks, support vector machines, random forests, etc.) to count crops, detect color and classify stress in various crops through images [6–9]. These recent literature demonstrates the impact traditional machine learning has on the future of agriculture. However, these methods are not without limitations. Using traditional approaches oftentimes, requires high quality images, constant lighting conditions, and fixed camera distances. These limitations act as a barrier to true HTP due to restricting the flow of information due to physical constraints. With advances in state-of-the-art deep learning techniques, these constraints are no longer binding. Robust models can be constructed to analyze crops in numerous variable conditions. This is seen in the current literature combining deep learning and HTP.

Image-based phenotyping and deep learning can broadly be labeled as an application area of computer vision. Traditional tasks include classifying single images, counting objects, and detecting objects. Common deep learning frameworks using U-Net, CenterNet, and ResNet-50 architectures have been applied to classify various fruits and vegetables from single images [10–14]. Other deep learning models using VGG-16 as a feature extractor and the “You Only Look Once” model have been used to count and detect leaves, sorghum heads, and corn kernels [15–20]. Using novel frameworks to count corn tassels, [21] combined convolutional neural networks (CNNs) and regression into a framework they named TasselNet. Additionally, open-access, high-quality, annotated datasets are being created and released to the public to engage researchers in applying their deep learning knowledge to agriculture [22, 23]. Indeed, these works showcase the promise deep learning has in providing large scale image analysis for HTP. Alone, advanced analytical techniques cannot directly mitigate the data collection process, but considering the end-to-end approach of data collection, data analysis, and scalable deployment, deep learning can efficiently aid this task. We point the interested reader to [24] where they provide a comprehensive review of deep learning in horticulture.

Corn is known to be one of the world’s most essential crops due to the number of products that it can create (e.g. flour, bio-fuels) [25]. Additionally, a large percentage of corn is used in livestock farming to feed pigs, cattle, and cows. The world’s reliance on corn cannot be understated. Aside from the manufacturing aspect, corn has a large impact on the United States’ economy. In 2019, it is estimated that the U.S. corn market contributed approximately $140 billion to its economy. It is evident that the agricultural community and the world must act to ensure the continued optimal production of corn. [26] states that by 2050, the world’s population will be approximately 9 billion. With the increase in population combined with the non-increasing arable land, changes will need to occur so that we can continue to optimize corn yield while utilizing fewer resources. Prior literature evokes deep learning algorithms learning from genetics, environment, and satellite imagery data to predict corn yield. However, these works do not belong to the HTP area since they only act as a way to approximate yield potential throughout the crop’s growing cycle [27, 28].

Our research objective is to accurately identify the number of corn stands from a specific area on the field with an image taken during the early phenological stages (VE to V6) [29]. Roughly these phenological stages refer to the visible leaves on the stem. For instance, VE (emergence) is the first phenological stage where the stem breaks through the soil. V1 is the appearance of the first leaf. V2 is the appearance of the second leaf, and VN is the appearance of the *n*-th leaf. From a practical perspective, estimating early stand count values allow for the establishment of a planting rate and, ultimately, yield potential. If the proper rate is planted, then farmers/breeders can estimate yield based on product by population. However, if the population is not there i.e. poor germination/ bad planter, then farmers can identify the issue and replant, or at the very least establish what the new yield will be. Having an estimate of yield prior to harvest then allows for key financial decision making tasks such as how much grain to hold, or when to sell. Knowing that the planting density is below its desired threshold enables farmers to decide how they want to best manage their corn to make up for the difference in planting rating (e.g. more fertilizer, more aggressive pesticide control, etc.). Traditionally, farmers perform stand count estimations manually. However, this process is time consuming, labor intensive, and susceptible to human error. Because of this, there is a reluctance to perform a stand count, ultimately, leaving farmers at a net loss for the corn yield. In the end, if there was an efficient, deployable, framework that enabled large scale phenotyping for early stage stand counting, farmers would have additional information to maximize their yield potential and, ultimately, their net profit for each planting season. Utilizing an image-based approach to this problem will allow for the timely estimation of stand counts and as well as a consistent measure for the quality of data.

Due to the need for efficient and effective HTP, this work presents a deep learning algorithm, named DeepStand, to alleviate labor intensive stand counting. The proposed method evokes a truncated VGG-16 network as a feature extractor backbone and combines multiple feature maps with varying scales to create a network robust against scale variation. This approach is similar to common methods in crowd counting where models are used to detect individual people in large crowds. Due to the similarity of these problems, we utilize a point density based approach for detecting the corn stands.

## Methodology

**Fig 1:**
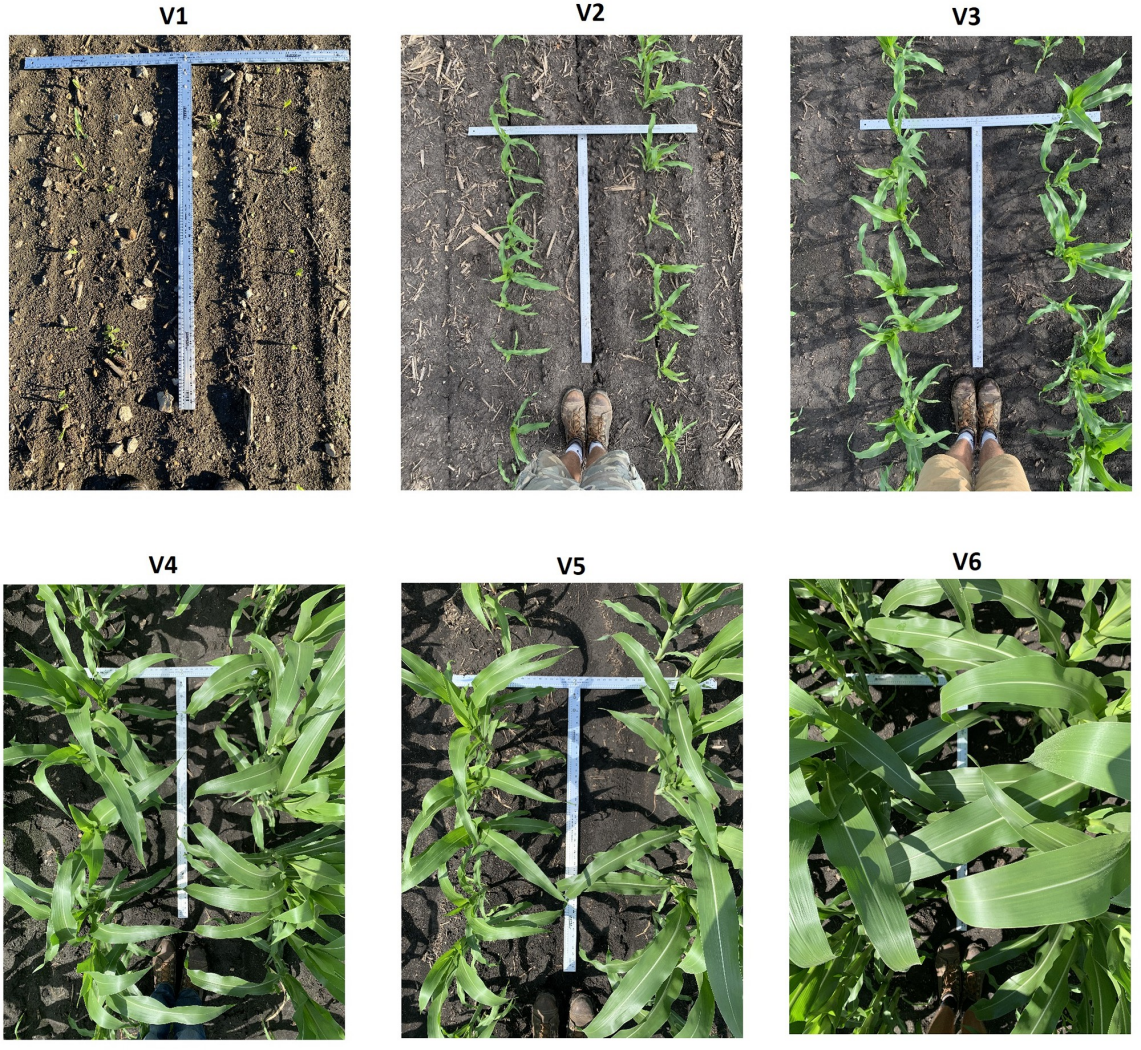
The images show corn stands at vegetative stages V1 to V6. The images include high scale variations, occlusions, and small distance between corn stands especially at stages V4 to V6.

Image-based corn stand counting is challenging compared to the counting tasks in other fields due to multiple factors, including occlusions, scale variations, and small distance between corn stands. Fig 1 shows the corn stands at different growing stages. This paper offers an innovative deep learning framework, DeepStand, to count the number of corn stands based on a single 180-degree image taken at 4-6 feet above the ground. It is worth noting that as corn progresses through its phenological stages, accurately counting the planting density becomes a difficult task for a computer due to the amount of overlapping leaves. However, from a pragmatic perspective, stand counting should be performed before V4 to ensure agronomists can act in a timely manner to mitigate any crop issues.

### Network architecture

Corn stand images usually include high scale variations and occluded corn stands by nearby corn leaves. Because of this, we design our algorithm to robust against these factors. The designed stand counting approach is inspired by architectures used for counting task in other fields such as crowd counting [30] and dense object counting [31].

**Fig 2:**
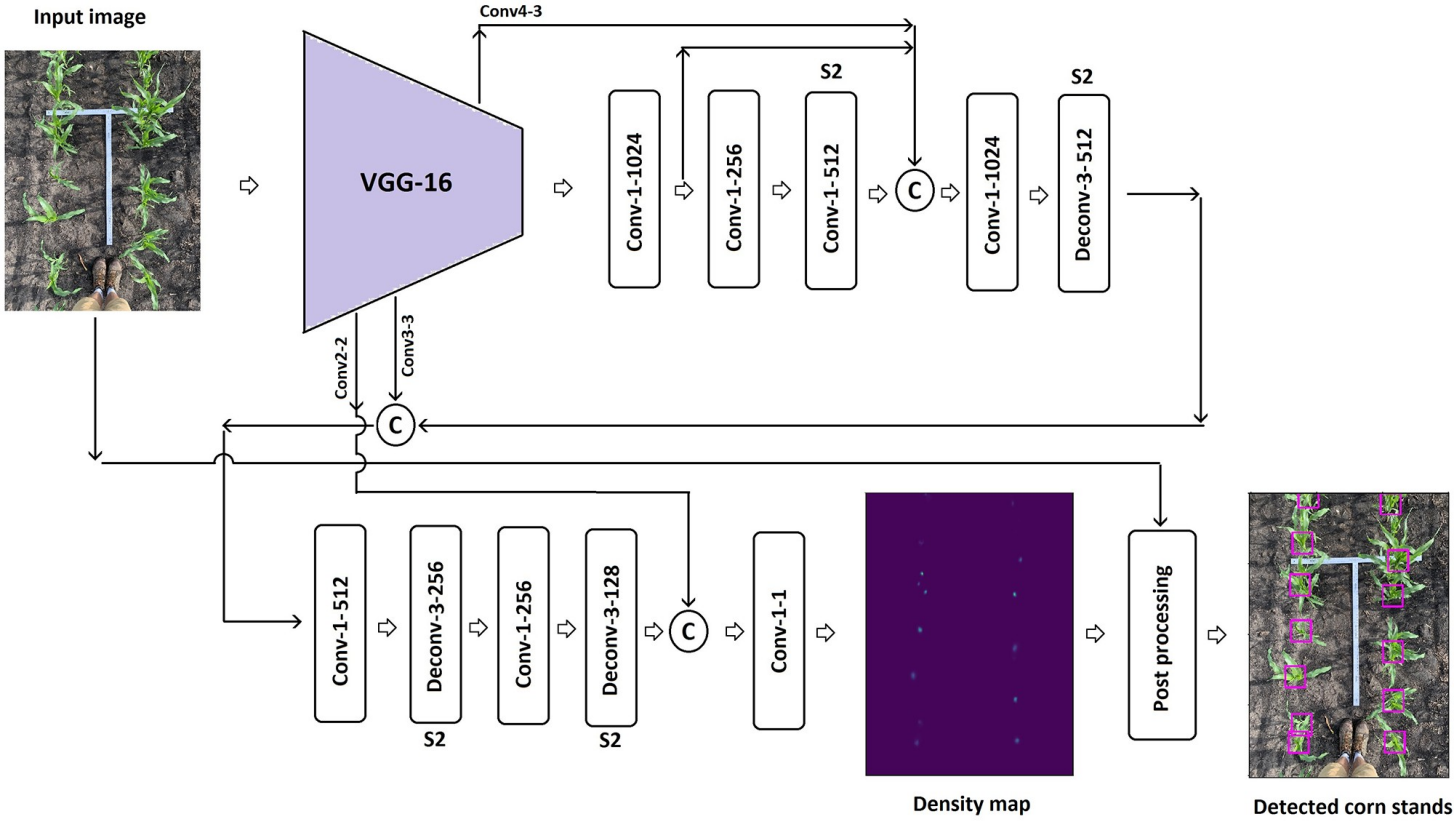
Overview of the proposed DeepStand architecture. The convolutional layers and deconvolutional layers parameters are represented as ‘Conv-(kernel size)-(number of filters)’ and ‘Deconv-(kernel size)-(number of filters)’, respectively. All layers have the stride of one except for the layers noted as “S2”, which have stride of two. The padding type is the ‘same’ except for the first deconvolutional layer for which we use ‘valid’ padding. © denotes matrix concatenation.

The architecture of the proposed network is outlined in Fig 2. Given an image as input, the proposed network generates a density map for the corn stands. The summation over the each density map provides the total number of corn stands in each image. DeepStand is a CNN-based density estimation method. We do not use other methods such as detection-based [32, 33] or regression-based [34–36] methods. This is because detection-based approaches such as such as single shot detection [37] or a detector via a sliding window [38] may not work well when applied to the scenes with occlusion and dense objects. Moreover, training these methods requires a large database of annotated images, which is not available to the public for corn stand counting. Additionally, we do not consider a regression-based approach because they disregard the spatial information in the input image. As such, these approaches do not know how much each image region contributes the final count.

We use a truncated VGG-16 [39] as a feature extraction backbone in our network. The truncated VGG-16 is composed of a fixed kernel size of 3 × 3 convolutional layers that extracts discriminative features from input image for further analysis of the network. We use the VGG-16 network backbone in our network mainly because of its good generalization ability to various computer vision applications such as object recognition and identification [40–43]. The truncated VGG-16 network includes all layers of the VGG-16 network with the exception of the fully connected layers and the last max-pooling layer. The truncated VGG-16 shrinks the input images’ resolution to the 1/8 of its original size. We increase input size of the truncated VGG-16 network from 224 × 224 to 300 × 300 in our proposed network to learn more fine-grained features and patterns from the input image to improve the performance of our DeepStand model [44].

Our ensure robustness against scale variations, our framework combines feature maps from multiple scales of the network. Other vision students have applied similar adaptive scaling architectures [45–47]. We concatenate feature maps with different spatial resolutions, by using zero padding to ensure each of the smaller feature maps have an equivalent dimension to the largest feature map. Finally to ensure that the output of our network produces an image that is the same size as the input, we apply three deconvolutional layers (transposed convolution) [48] with stride of two for upsampling.

Finally, we do a post processing on the predicted density map to visualization a bounding box for each detected corn stand. The post processing includes the following steps: (1) perform thresholding on the approximated density map to ignore negligible areas, (2) find peak coordinates on the density map as the center location of corn stands, (3) draw a bounding box around each corn stand, and (4) remove overlapping bounding boxes by a non-maximum suppression. The above-mentioned post processing has a very low computational cost and does not increase the inference time.

### Loss function

Let *I*_*i*_, *D*_*i*_, Θ, *F*(*I*_*i*_, Θ), and *N* denote *i*th image, the ground truth density map of the *i*th image, network parameters, the predicted density map of the *i*th image, and the number of input images, respectively. As such, we define our network loss function as follows:

$$\begin{array}{c} L\left(\Theta \right)=\frac{1}{N}\sum_{i=1}^{N}{\left\|F\left(I_{i},\Theta \right)-D_{i}\right\|}_{2}^{2} \end{array}$$ (1)

The Euclidean loss measures estimation error at the pixel level and is commonly applied in the computer vision domain [42, 49, 50].

## Experiments and results

To begin, we explain the dataset, data augmentation techniques, evaluation metrics, and training procedure. Then, we report a numeric comparision between DeepStand with other cutting-edge competing methods. All experiments were conducted with an NVIDIA Tesla V100 SXM2 GPU and Tensorflow [51]

### Data

#### Ground truth density maps generation

To compute ground truth density maps, we train the network parameters mimicking the process outlined by [30]. Given a single corn stand at pixel location *x*_*i*_, we define a delta function *δ*(*x* − *x*_*i*_). Therefore, given an image with *M* annotated corn stands provides the following the ground truth output:

$$\begin{array}{c} H\left(x\right)=\sum_{i=1}^{M}\delta \left(x-x_{i}\right) \end{array}$$ (2)

Following the ground truth output, we construct the density map *D*(*x*) by convolving a Gaussian kernel with *H*(*x*) with a standard deviation *σ*, where *k*-nearest neighboring is used to compute the standard deviation. By taking the sum over the entire density map, we have the total number of corn stands present in the image.

$$\begin{array}{c} D\left(x\right)=\sum_{i=1}^{M}\delta \left(x-x_{i}\right)*G_{\sigma }\left(x\right) \end{array}$$ (3)

#### Stand count data

This section presents the procedure to prepare sufficient data to train and evaluate our proposed method. Our original dataset includes 394 images of corn stands at growing stages V1 to V6 with a fixed size of 1024 × 768 taken at 4-6 feet above the ground. This includes a total of 6,154 total stands across all images. We used a publicly available tool called LabelMe to annotate all images [52]. That is, the authors manually identified and counted each of the 6,154 corn using the LabelMe annotation tool to be used as our ground truth. The following Table 1 provides the summary of our dataset.

**Table 1:** The corn stand dataset summary. Minimum, Maximum, and Average represent the summary number of corn stands across all 394 images.

Number of Images	Resolution	Min	Max	Avg	Total
394	1024 × 768	5	31	15.62	6154

For our training-test split, we used an 80-20 split at random. That is, we designated 20% of the images (80 images) for testing and used the remaining images for training (314 images). We perform data augmentation in the training dataset to generate additional observations to train DeepStand to ensure our method is robust against scale variation. To perform augmentation, we created a multi-scale pyramidal representation for each image in our training set [53]. The multi-scale pyramidal representation of images includes scales ranging from 0.4 to 1.3 with a step-size increment of 0.1, times the original image dimension. Next, 300 × 300 image patches are cropped at random locations followed by randomly flipping and adding Gaussian noise.

### Evaluation metrics

We use standard evaluation metrics, Mean Absolute Error (MAE), Root Mean Squared Error (RMSE), mean absolute percentage error (MAPE), and bias to measure the counting performance of the proposed method. Generally, MAE indicate the accuracy of the results and RMSE measures the robustness. MAPE measures the accuracy in terms of error percentage, and bias shows if the model tends to overestimate or underestimate the actual counts. These four metrics are defined as follows:

$$\begin{array}{c} MAE=\frac{1}{N}\sum_{i=1}^{N}\left|C_{i}^{pred}-C_{i}^{GT}\right| \end{array}$$ (4)

$$\begin{array}{c} RMSE=\sqrt{\frac{1}{N}\sum_{i=1}^{N}{\left|C_{i}^{pred}-C_{i}^{GT}\right|}^{2}} \end{array}$$ (5)

$$\begin{array}{c} MAPE=\frac{1}{N}\sum_{i=1}^{N}\left|\frac{C_{i}^{GT}-C_{i}^{pred}}{C_{i}^{GT}}\right|\times 100 \end{array}$$ (6)

$$\begin{array}{c} Bias=\frac{1}{N}\sum_{i=1}^{N}\left(C_{i}^{pred}-C_{i}^{GT}\right) \end{array}$$ (7)

where *N* is the number of test images, $C_{i}^{pred}$ and $C_{i}^{GT}$ are the estimated count and the ground truth count corresponding to the *i*th test image.

### Training hyperparameter

We train our DeepStand network from end-to-end. Our deep learning network parameters are initialized with Xavier initialization [54]. Adam optimizer [55] with learning rate of 3e-4 and 24 as our mini-batch size are used to minimize the network loss function from Eq (1). The learning rate is gradually decayed to 25e-6 during the training process. The network is trained 80,000 iterations on 93,258 image patches generated following the data augmentation procedure in.

### Comparison with state-of-the-art

We implement five state-of-the-art models to evaluate the efficiency of DeepStand. These five models were designed for crowd counting applications. However, due to the density of our corn stands, these approaches are also applicable for our use case. The following benchmark models include:

**CSRNet**: Utilizes a fully convolutional framework that includes a truncated VGG-16 network feature extractor on the front-end with dilated convolutional layers to estimate the density map [43].
**SaCNN**: Applies a scale-adaptive convolutional neural network to handle scale and perspective change in images. The SaCNN uses a backbone similar to a VGG-16 architecture for feature extraction and then combines feature maps from different layers to ensure robustness to scale variations [56].
**MSCNN**: Extracts scale-relevant features using a multi-scale CNN network. The MSCNN network consists of multiple Inception-like [57] modules for multi-scale feature extraction [58].
**CrowdNet**: Uses a multi-column network architecture in two parts. The first is multiple CNN layers and the second is a shallow network to predict density map. The deep CNN has a VGG-like network architecture and the shallow CNN includes three convolutional layers [30].
**DeepCrowd**: Employs a regression-based method to establish a function from image patches to the total object count. The DeepCrowd network architecture applies a combinations of CNNs and fully connected layers [35].

### Results

This section reports the numerical evaluation results and compares DeepStand with other cutting-edge algorithms for the task of corn stand counting. After having trained all methods, we assessed their prediction accuracy on the independent hold-out dataset which includes 80 images of corn stands from growing stages V1 to V6. Table 2 illustrate the stand counting performance of DeepStand and comparison methods on the test data with respect to MAE and RMSE evaluation metrics.

**Table 2:** The counting performances of the proposed and comparison methods on the test data.

Method	MAE	RMSE	MAPE(%)	Bias
CSRNet [43]	2.23	2.84	16.18	-0.78
SaCNN [56]	4.38	5.25	31.92	-4.33
MSCNN [58]	2.26	2.65	14.65	0.53
CrowdNet [30]	4.49	5.53	32.23	4.86
DeepCrowd [35]	2.61	3.13	17.67	**0.33**
DeepStand (our proposed)	**1.73**	**2.46**	**12.25**	0.58

Table 2 illustrates that our proposed stand counting method performs competitively to the other methods to varying extents. The MSCNN has a comparable performance with CSRNet demonstrating that they are the more accurate and robust benchmark algorithms. The main reason for the good performance of CSRNet is because of utilizing dilated convolutional layers to perform an estimation of the density maps. The performance of MSCNN can be attributed to the robustness against scale variations by the use of multi-scale features. Using a different approach, we see that the DeepCrowd method outperforms the SaCNN and the CrowdNet algorithm due to its regression framework. We believe DeepStand numerically outperforms other state-of-the-art methods because (1) our proposed method combines feature maps from multiple scales of the network to deal with scale variation, and (2) the use of deconvolution layers for up-sampling the feature maps increases the quality of the predicted density maps.

Even though the counting performance of the CSRnet, MSCNN, DeepCrowd, and SaCNN are respectable, the limitations are apparent when an whole image is used as input as opposed to mini-patches. As a result, an input image should be cropped into distinct partitions and fed to these methods to increase their inference time. Unlike competing algorithms, the inference time of DeepStand and CrowdNet is considerably faster because these two algorithms accepts an entire image as input and provides predictions with a single forward path.

**Fig 3:**
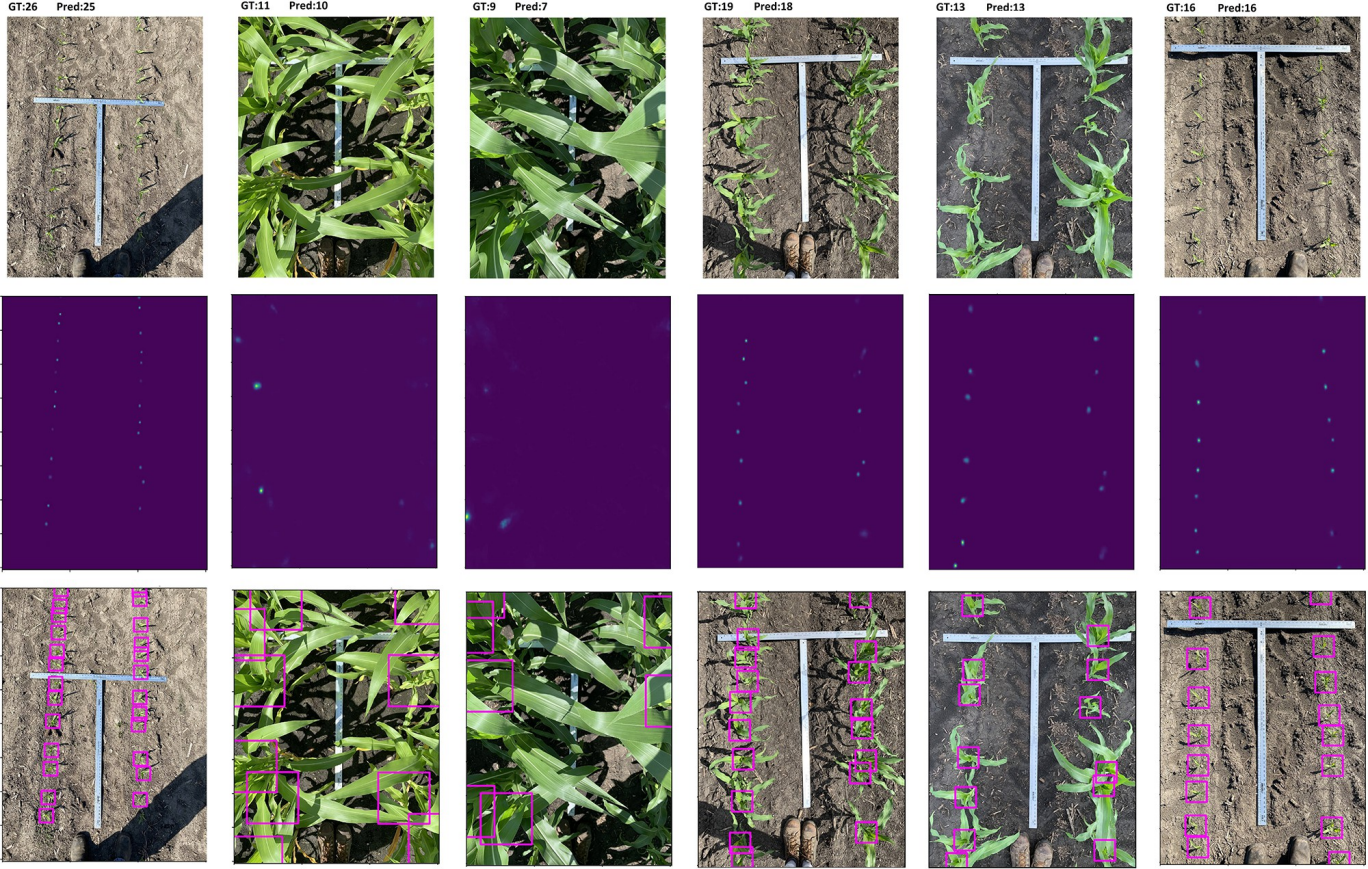
A visual illustration of the localization of our DeepStand model. The three rows represent the input images, density map estimations, and identified corn stand, respectively. GT and Pred represent the ground truth number of stands and the predicted number of corn stands, respectively. The figure is best viewed in color.

Fig 3 visualizes some stand counting results of our proposed method including original image, predicted density map, and the detected corn stands in an image.

In order to see how the growing stages (VE to V6) affect the counting performance of the proposed method, we divide the test data based on the growing stages into three classes of VE-V1, V2-V4, and V5-V6 and report the counting performances of the proposed and comparison methods on these classes. Table 3 summarizes our numerical results. We notice that our proposed method has a consistently low error across all growing stages which indicates the robustness of our proposed method. The results also indicate that the highest counting error of all methods except MSCNN belong to the stage V5-V6 due to the occlusion and background clutter.

**Table 3:** The performances DeepStand and comparison methods on the test data across different growing stages. The number of samples in test data across stages VE-V1, V2-V4, and V5-V6 are 14, 36, 30, respectively.

Method	Growing stage											
	VE-V1				V2-V4				V5-V6			
	MAE	RMSE	MAPE(%)	Bias	MAE	RMSE	MAPE(%)	Bias	MAE	RMSE	MAPE(%)	Bias
CSRNet [43]	2.34	3.12	13.96	-2.24	1.51	**1.89**	8.62	1.10	3.04	3.56	26.28	-2.32
SaCNN [56]	5.87	6.28	35.88	-5.72	2.25	2.49	13.34	-2.19	6.24	6.90	52.38	-6.24
MSCNN [58]	2.72	2.98	16.67	-1.43	2.37	2.76	13.17	1.98	**1.91**	**2.32**	**15.48**	**-0.29**
CrowdNet [30]	3.71	4.88	20.41	3.57	4.43	4.94	24.45	4.43	5.97	6.41	47.05	5.97
DeepCrowd [35]	3.23	3.67	20.25	-2.09	2.57	2.91	14.59	2.49	2.39	3.11	20.15	-1.12
DeepStand (our proposed)	**1.0**	**1.31**	**5.89**	**-0.29**	**1.39**	1.94	**7.9**	**0.0**	2.47	3.30	20.42	1.67

## Discussion and conclusion

Historically, intensive manual labor, time, and domain expertise were required to accurately record crop phenotypes—referred to as the phenotyping bottleneck. Given the need for efficient scalable crop phenotyping due to resource limitations, organizations have adapted to utilizing image capturing devices. That is, organizations have progressed towards innovative technologies for large scale phenotyping, also known as high-throughput phenotyping. These new mechanisms allow for the analysis and identification of traits through the use of advanced analytical methods. However, there is still work to be performed to maximize efficiency namely through the development of computer vision algorithms to accuracy analyze images.

Our manuscript provides a novel deep learning based algorithm we call DeepStand to count corn stands from images. This architecture adopts a truncated VGG-16 network as a backbone feature extractor and merges multiple feature maps with different scales to make the network robust against scale variation. Finally, DeepStand uses a set of deconvolutional layers as a back-end to up-sample the network output. From extensive experimental results, we illustrate that DeepStand can efficiently and effectively identify and detect corn stands regardless of the image scale and the lighting condition. Further, we implemented five state-of-the-art competing algorithms and showed that DeepStand is competitive and, in all but three cases, outperforms those models.

Our numerical results provide hope towards the future of modernizing agriculture to enable true high-throughput phenotyping and, ultimately, better enabling agronomists with information to enhance decision making. Indeed, as shown in Table 3, DeepStand provides accurate stand estimates for phenological stages VE-V4, where farmers traditionally desire stand counts. Simply due to the difficulty of overlapping leaves and background clutter at this moment we are not able to construct a model which can handle stages beyond V4. However, from a practical perspective, this is inessential. With the continued development of computing resources and algorithms, we hope to one day revisit this problem to construct a robust model that will work no matter the growth stage.

Although this work focuses on counting corn stands, a similar approach may be generalized to similar yet different applications such as detecting sorghum heads, counting wheat heads, detecting weeds, identifying planting gaps, etc. The open challenges facing high-throughput phenotyping are vast and require the joint collaboration of machine learning researchers to be able to solve these problems.

## References

[1] Furbank RT, Tester M. Phenomics-technologies to relieve the phenotyping bottleneck. Trends in plant science. 2011;16(12):635–644. doi: 10.1016/j.tplants.2011.09.005 22074787

[2] Araus JL, Kefauver SC, Zaman-Allah M, Olsen MS, Cairns JE. Translating high-throughput phenotyping into genetic gain. Trends in plant science. 2018;23(5):451–466. doi: 10.1016/j.tplants.2018.02.001 29555431 PMC5931794

[3] McWilliams DA, Berglund DR, Endres G. Corn growth and management quick guide. 1999.

[4] Mogili UR, Deepak B. Review on application of drone systems in precision agriculture. Procedia computer science. 2018;133:502–509. doi: 10.1016/j.procs.2018.07.063

[5] Kulbacki M, Segen J, Knieć W, Klempous R, Kluwak K, Nikodem J, et al. Survey of drones for agriculture automation from planting to harvest. In: 2018 IEEE 22nd International Conference on Intelligent Engineering Systems (INES). IEEE; 2018. p. 000353-000358.

[6] Naik HS, Zhang J, Lofquist A, Assefa T, Sarkar S, Ackerman D, et al. A real-time phenotyping framework using machine learning for plant stress severity rating in soybean. Plant methods. 2017;13(1):23. doi: 10.1186/s13007-017-0173-7 28405214 PMC5385078

[7] Rajan P, Radhakrishnan B, Suresh LP. Detection and classification of pests from crop images using support vector machine. In: 2016 international conference on emerging technological trends (ICETT). IEEE; 2016. p. 1-6.

[8] Owomugisha G, Mwebaze E. Machine learning for plant disease incidence and severity measurements from leaf images. In: 2016 15th IEEE international conference on machine learning and applications (ICMLA). IEEE; 2016. p. 158-163.

[9] Torres-Sánchez J, López-Granados F, Serrano N, Arquero O, Peña JM. High-throughput 3-D monitoring of agricultural-tree plantations with unmanned aerial vehicle (UAV) technology. PloS one. 2015;10(6):e0130479. doi: 10.1371/journal.pone.0130479 26107174 PMC4479442

[10] Choi S, Lee S, Kang Y, Choi DY, Choi J. Use of Unmanned Aerial Vehicle Imagery and Deep Learning UNet to Classification Upland Crop in Small Scale Agricultural Land. Journal of the Korean Society of Surveying, Geodesy, Photogrammetry and Cartography. 2020;38(6):671–679.

[11] Lin Z, Guo W. Cotton Stand Counting from Unmanned Aerial System Imagery Using MobileNet and CenterNet Deep Learning Models. Remote Sensing. 2021;13(14):2822. doi: 10.3390/rs13142822

[12] Hu WJ, Fan J, Du YX, Li BS, Xiong N, Bekkering E. MDFC-ResNet: An Agricultural IoT System to Accurately Recognize Crop Diseases. IEEE Access. 2020;8:115287–115298. doi: 10.1109/ACCESS.2020.3001237

[13] Khaki S, Wang L, Archontoulis SV. A CNN-RNN Framework for Crop Yield Prediction. Frontiers in Plant Science. 2019;10. doi: 10.3389/fpls.2019.01750 32038699 PMC6993602

[14] Khaki S, Wang L. Crop Yield Prediction Using Deep Neural Networks. Frontiers in Plant Science. 2019;10:621. doi: 10.3389/fpls.2019.00621 31191564 PMC6540942

[15] Kumar DAS, AR D, Babu LG, Suresh DG, et al. Smart Agriculture Robo With Leaf Diseases Detection Using IOT. European Journal of Molecular & Clinical Medicine. 2022;7(11):2462–2469.

[16] Zhao W, Yamada W, Li T, Digman M, Runge T. Augmenting Crop Detection for Precision Agriculture with Deep Visual Transfer Learning—A Case Study of Bale Detection. Remote Sensing. 2021;13(1):23. doi: 10.3390/rs13010023

[17] Mosley L, Pham H, Bansal Y, Hare E. Image-Based Sorghum Head Counting When You Only Look Once. arXiv preprint arXiv:200911929. 2020.

[18] Khaki S, Pham H, Han Y, Kuhl A, Kent W, Wang L. Deepcorn: A semi-supervised deep learning method for high-throughput image-based corn kernel counting and yield estimation. Knowledge-Based Systems. 2021;218:106874. doi: 10.1016/j.knosys.2021.106874

[19] Olsen PA, Ramamurthy KN, Ribera J, Chen Y, Thompson AM, Luss R, et al. Detecting and counting panicles in sorghum images. In: 2018 IEEE 5th International Conference on Data Science and Advanced Analytics (DSAA). IEEE; 2018. p. 400-409.

[20] Redmon J, Divvala S, Girshick R, Farhadi A. You only look once: Unified, real-time object detection. In: Proceedings of the IEEE conference on computer vision and pattern recognition; 2016. p. 779-788.

[21] Lu H, Cao Z. Tasselnetv2+: A fast implementation for high-throughput plant counting from high-resolution RGB imagery. Frontiers in plant science. 2020;11:1929. doi: 10.3389/fpls.2020.541960 33365037 PMC7750361

[22] Chiu MT, Xu X, Wei Y, Huang Z, Schwing AG, Brunner R, et al. Agriculture-vision: A large aerial image database for agricultural pattern analysis. In: Proceedings of the IEEE/CVF Conference on Computer Vision and Pattern Recognition; 2020. p. 2828-2838.

[23] Lu Y, Young S. A survey of public datasets for computer vision tasks in precision agriculture. Computers and Electronics in Agriculture. 2020;178:105760. doi: 10.1016/j.compag.2020.105760

[24] Yang B, Xu Y. Applications of deep-learning approaches in horticultural research: a review. Horticulture Research. 2021;8(1):1–31. doi: 10.1038/s41438-021-00560-9 34059657 PMC8167084

[25] Tyndall JC, Berg EJ, Colletti JP. Corn stover as a biofuel feedstock in Iowa’s bio-economy: an Iowa farmer survey. Biomass and bioenergy. 2011;35(4):1485–1495. doi: 10.1016/j.biombioe.2010.08.049

[26] Tripathi AD, Mishra R, Maurya KK, Singh RB, Wilson DW. Estimates for world population and global food availability for global health. In: The role of functional food security in global health. Elsevier; 2019. p. 3–24.

[27] Khaki S, Khalilzadeh Z, Wang L. Classification of crop tolerance to heat and drought—a deep convolutional neural networks approach. Agronomy. 2019;9(12):833. doi: 10.3390/agronomy9120833

[28] Khaki S, Khalilzadeh Z, Wang L. Predicting yield performance of parents in plant breeding: A neural collaborative filtering approach. Plos one. 2020;15(5):e0233382. doi: 10.1371/journal.pone.0233382 32437473 PMC7241707

[29] Anandhi A. Growing degree days-Ecosystem indicator for changing diurnal temperatures and their impact on corn growth stages in Kansas. Ecological Indicators. 2016;61:149–158. doi: 10.1016/j.ecolind.2015.08.023

[30] Boominathan L, Kruthiventi SS, Babu RV. Crowdnet: A deep convolutional network for dense crowd counting. In: Proceedings of the 24th ACM international conference on Multimedia; 2016. p. 640-644.

[31] Qiu H, Ma Y, Li Z, Liu S, Sun J. Borderdet: Border feature for dense object detection. In: European Conference on Computer Vision. Springer; 2020. p. 549-564.

[32] Li M, Zhang Z, Huang K, Tan T. Estimating the number of people in crowded scenes by mid based foreground segmentation and head-shoulder detection. In: 2008 19th international conference on pattern recognition. IEEE; 2008. p. 1-4.

[33] Zeng C, Ma H. Robust head-shoulder detection by pca-based multilevel hog-lbp detector for people counting. In: 2010 20th International Conference on Pattern Recognition. IEEE; 2010. p. 2069-2072.

[34] Li R, Wang R, Xie C, Chen H, Long Q, Liu L, et al. A multi-branch convolutional neural network with density map for aphid counting. Biosystems Engineering. 2022;213:148–161. doi: 10.1016/j.biosystemseng.2021.11.020

[35] Wang C, Zhang H, Yang L, Liu S, Cao X. Deep people counting in extremely dense crowds. In: Proceedings of the 23rd ACM international conference on Multimedia; 2015. p. 1299-1302.

[36] Idrees H, Saleemi I, Seibert C, Shah M. Multi-source multi-scale counting in extremely dense crowd images. In: Proceedings of the IEEE conference on computer vision and pattern recognition; 2013. p. 2547-2554.

[37] Phillips ZF, Chen M, Waller L. Single-shot quantitative phase microscopy with color-multiplexed differential phase contrast (cDPC). PloS one. 2017;12(2):e0171228. doi: 10.1371/journal.pone.0171228 28152023 PMC5289592

[38] Khaki S, Pham H, Han Y, Kuhl A, Kent W, Wang L. Convolutional Neural Networks for Image-Based Corn Kernel Detection and Counting. Sensors. 2020;20(9):2721. doi: 10.3390/s20092721 32397598 PMC7249160

[39] Gopalakrishnan K, Khaitan SK, Choudhary A, Agrawal A. Deep convolutional neural networks with transfer learning for computer vision-based data-driven pavement distress detection. Construction and building materials. 2017;157:322–330. doi: 10.1016/j.conbuildmat.2017.09.110

[40] Paymode AS, Malode VB. Transfer learning for multi-crop leaf disease image classification using convolutional neural networks VGG. Artificial Intelligence in Agriculture. 2022.

[41] Yarak K, Witayangkurn A, Kritiyutanont K, Arunplod C, Shibasaki R. Oil Palm Tree Detection and Health Classification on High-Resolution Imagery Using Deep Learning. Agriculture. 2021;11(2):183. doi: 10.3390/agriculture11020183

[42] Gao G, Liu Q, Wang Y. Counting dense objects in remote sensing images. In: ICASSP 2020-2020 IEEE International Conference on Acoustics, Speech and Signal Processing (ICASSP). IEEE; 2020. p. 4137-4141.

[43] Li Y, Zhang X, Chen D. Csrnet: Dilated convolutional neural networks for understanding the highly congested scenes. In: Proceedings of the IEEE conference on computer vision and pattern recognition; 2018. p. 1091-1100.

[44] Sitaula C, Hossain MB. Attention-based VGG-16 model for COVID-19 chest X-ray image classification. Applied Intelligence. 2021;51(5):2850–2863. doi: 10.1007/s10489-020-02055-x 34764568 PMC7669488

[45] Ji S, Wei S, Lu M. A scale robust convolutional neural network for automatic building extraction from aerial and satellite imagery. International journal of remote sensing. 2019;40(9):3308–3322. doi: 10.1080/01431161.2018.1528024

[46] Bai H, Wen S, Gary Chan SH. Crowd counting on images with scale variation and isolated clusters. In: Proceedings of the IEEE International Conference on Computer Vision Workshops; 2019. p. 0-0.

[47] Ronneberger O, Fischer P, Brox T. U-net: Convolutional networks for biomedical image segmentation. In: International Conference on Medical image computing and computer-assisted intervention. Springer; 2015. p. 234-241.

[48] Dumoulin V, Visin F. A guide to convolution arithmetic for deep learning; 2016.

[49] Lian D, Li J, Zheng J, Luo W, Gao S. Density map regression guided detection network for rgb-d crowd counting and localization. In: Proceedings of the IEEE Conference on Computer Vision and Pattern Recognition; 2019. p. 1821-1830.

[50] Eisenschtat A, Wolf L. Linking image and text with 2-way nets. In: Proceedings of the IEEE conference on computer vision and pattern recognition; 2017. p. 4601-4611.

[51] Dillon JV, Langmore I, Tran D, Brevdo E, Vasudevan S, Moore D, et al. Tensorflow distributions. arXiv preprint arXiv:171110604. 2017.

[52] Russell BC, Torralba A, Murphy KP, Freeman WT. LabelMe: a database and web-based tool for image annotation. International journal of computer vision. 2008;77(1):157–173. doi: 10.1007/s11263-007-0090-8

[53] Martinel N, Luca Foresti G, Micheloni C. Aggregating deep pyramidal representations for person re-identification. In: Proceedings of the IEEE/CVF Conference on Computer Vision and Pattern Recognition Workshops; 2019. p. 0-0.

[54] Sun W, Su F, Wang L. Improving deep neural networks with multi-layer maxout networks and a novel initialization method. Neurocomputing. 2018;278:34–40. doi: 10.1016/j.neucom.2017.05.103

[55] Khaire UM, Dhanalakshmi R. High-dimensional microarray dataset classification using an improved adam optimizer (iAdam). Journal of Ambient Intelligence and Humanized Computing. 2020;11(11):5187–5204. doi: 10.1007/s12652-020-01832-3

[56] Zhang L, Shi M, Chen Q. Crowd counting via scale-adaptive convolutional neural network. In: 2018 IEEE Winter Conference on Applications of Computer Vision (WACV). IEEE; 2018. p. 1113-1121.

[57] Szegedy C, Liu W, Jia Y, Sermanet P, Reed S, Anguelov D, et al. Going deeper with convolutions. In: Proceedings of the IEEE conference on computer vision and pattern recognition; 2015. p. 1-9.

[58] Zeng L, Xu X, Cai B, Qiu S, Zhang T. Multi-scale convolutional neural networks for crowd counting. In: 2017 IEEE International Conference on Image Processing (ICIP). IEEE; 2017. p. 465-469.

